# CRISPR/Cas9‐mediated generation of non‐motile mutants to improve the harvesting efficiency of mass‐cultivated *Euglena gracilis*

**DOI:** 10.1111/pbi.13904

**Published:** 2022-09-08

**Authors:** Marumi Ishikawa, Toshihisa Nomura, Shun Tamaki, Kazunari Ozasa, Tomoko Suzuki, Kiminori Toyooka, Kikue Hirota, Koji Yamada, Kengo Suzuki, Keiichi Mochida

**Affiliations:** ^1^ Microalgae Production Control Technology Laboratory, RIKEN Baton Zone Program, RIKEN Cluster for Science Technology and Innovation Hub Yokohama Japan; ^2^ Bioproductivity Informatics Research Team RIKEN Center for Sustainable Resource Science Yokohama Japan; ^3^ Advanced Laser Processing Research Team RIKEN Center for Advanced Photonics Wako Japan; ^4^ Mass Spectrometry and Microscopy Unit, Technology Platform Division RIKEN Center for Sustainable Resource Science Kanagawa Japan; ^5^ Center for Gene Research Nagoya University Aichi Japan; ^6^ euglena Co., Ltd. Tokyo Japan; ^7^ Kihara Institute for Biological Research Yokohama City University Yokohama Japan; ^8^ Graduate School of Nanobioscience Yokohama City University Yokohama Japan; ^9^ School of Information and Data Sciences Nagasaki University Nagasaki Japan

**Keywords:** *Euglena gracilis*, harvesting efficiency, Cas9 ribonucleoprotein (RNP)‐based genome editing, *Bardet–Biedl syndrome (BBS)* genes

Non‐motile photosynthetic flagellates sediment well and thus can be harvested more easily under mass cultivation (Brennan and Owende, 2010). However, a precise genetic manipulation of microalgal motility remains challenging. The nutrient‐rich microalga *Euglena gracilis* is widely used in the food, cosmetics and feed industries (Suzuki, 2017). This alga accumulates paramylon, a crystalline β‐1,3‐glucan with multiple industrial applications (Harada *et al*., 2020). Under hypoxia, *E. gracilis* degrades paramylon to generate energy and converts it into wax esters, primarily myristic acid (C14:0) and myristyl alcohol (C14:0) (Inui *et al*., 2017), which are a potential source of jet biofuel. To improve the harvesting efficiency of *E. gracilis*, a non‐motile mutant ($M_{3}^{-}\text{ZFeL}$) was generated using Fe‐ion beam irradiation. However, $M_{3}^{-}\text{ZFeL}$ grew slower and produced less lipids than the wild type (WT), suggesting that the mutagenesis caused undesirable side mutations (Muramatsu *et al*., 2020). Although flagellar mutants with motility defects have been identified in model organisms such as *Chlamydomonas reinhardtii*, *Trypanosoma brucei* and *Caenorhabditis elegans* (Kamiya *et al*., 1991; Wingfield *et al*., 2018), a precise manipulation of motility in *E. gracilis* remains to be explored, despite its industrial usefulness.

Here, using our Cas9 ribonucleoprotein (RNP)‐based genome‐editing technique (Nomura *et al*., 2019), we targeted the *Bardet–Biedl syndrome (BBS)* genes in *E. gracilis* to generate non‐motile strains. We identified the genes encoding proteins homologous to *Caenorhabditis elegans* BBS‐7 (NP_499585.1; tblastn *E* value 7e–72) or BBS‐8 (NP_504711.2; tblastn *E* value 7e–123). These BBSome components are associated with the intraflagellar transport of particles and mediate the trafficking of cilium/flagellum membrane proteins in eukaryotic cells (Hammond *et al*., 2021; Nakayama and Katoh, 2018; Wingfield *et al*., 2018). We designed two pairs of single guide RNAs (sgRNAs) that target different regions of *EgBBS7* and *EgBBS8* and introduced Cas9–sgRNA RNPs into *E. gracilis* cells by electroporation (Method S1, Table S1).

Assessing the motility of the electroporated *E. gracilis* cells, we identified four sets of target sequences and protospacer adjacent motifs (PAMs), which are useful for inducing the stable non‐motility phenotype in *E. gracilis* (*EgBBS7*‐A and *EgBBS7*‐B; and *EgBBS8*‐B and *EgBBS8*‐D). To establish clonal *bbs* strains, we isolated two cell lines each for *bbs7* and *bbs8* (Method S1). PCR‐based genotyping detected 500–1500 bp deletions within the *EgBBS7* and *EgBBS8* target regions in these strains (Method S2, Figure 1a,b), and Sanger sequencing verified that large genomic regions were deleted, including intronic regions (Figure 1c,d). When isolated single cells were cultured, all eight *bbs* mutant strains flocculated, whereas the WT spread (Figure 1e). We confirmed the non‐flagellar phenotype of the mutant strains by scanning electron microscopy (Method S3, Figure 1f,g) and their non‐motility by a trace momentum assay that quantifies swimming motion (Method S4, Figure 1h,i). The results indicate that *EgBBS7* and *EgBBS8* contribute to forming a full‐length flagellum in *E. gracilis* and are thus required for motility.

**Figure 1:**
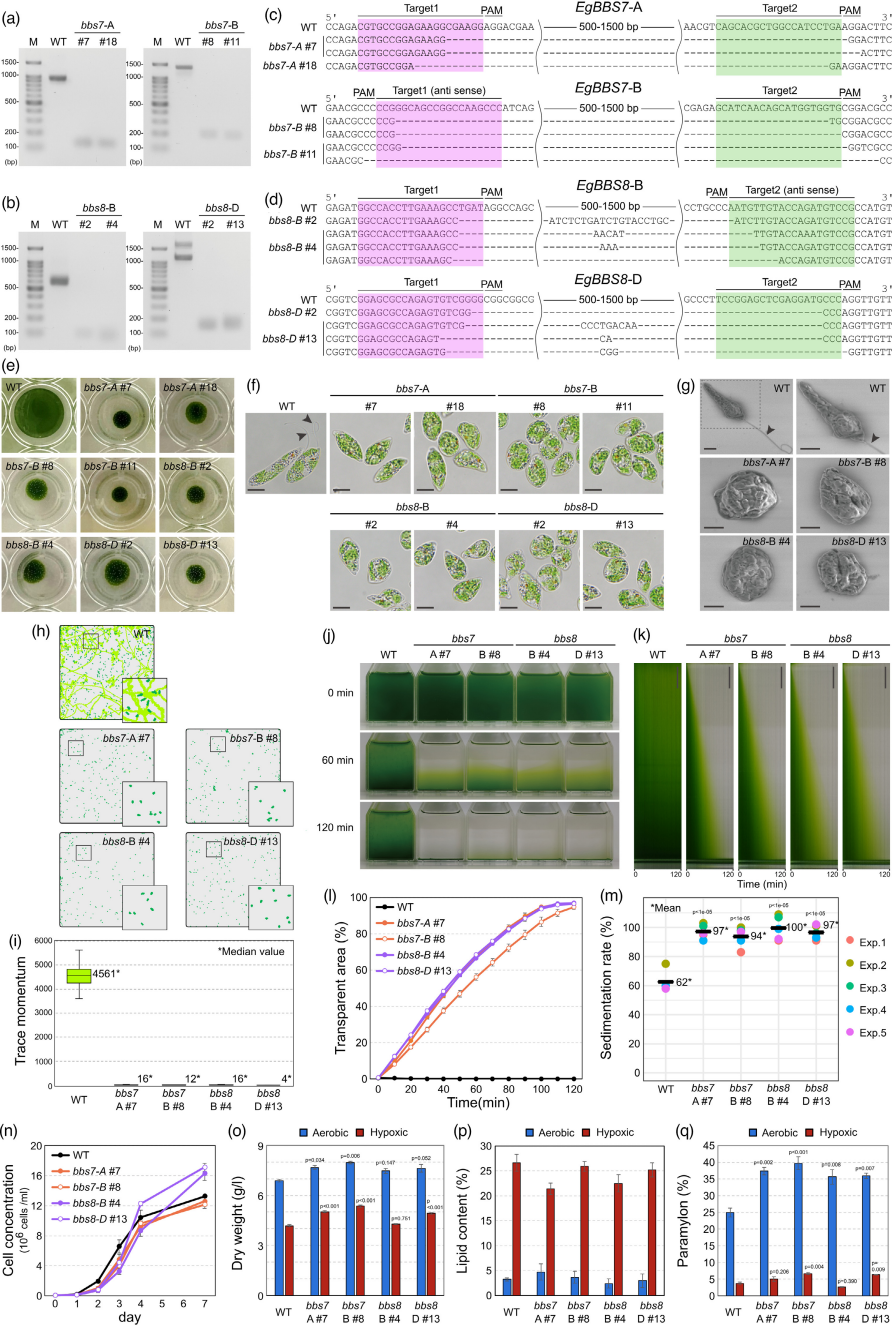
Generating non‐motile *Euglena gracilis* mutants by creating CRISPR/Cas9‐targeted deletions in *EgBBS7* and *EgBBS8*. (a, b) Detection of the truncated PCR fragments of *EgBBS7* (a) and *EgBBS8* (b). (c, d) Alignment of representative mutated sequences in truncated PCR fragments of *EgBBS7* (c) and *EgBBS8* (d) vs. WT. (e) Colony formation by isolated *bbs7* and *bbs8* cells. (f, g) Micrographs of the *bbs7* and *bbs8* mutants cultured in KH medium for 4 days taken by light microscopy (f, Scale bars, 10 μm) and scanning electron microscopy (g, Scale bars, 5 μm). Arrowheads indicate the flagellum. (h, i) Trace momentum assay. Green dots and light green lines indicate cells and their trajectory, respectively. (h) Trace momentum was calculated based on the total area of light green lines obtained at 450 time points in 10 min (i). (j–m) Sedimentation analysis. Time‐lapse observation of gravitational sedimentation (j) and 2D kymographs representing sedimentation speed (k, scale bars, 5 mm). Transparent areas were analysed in images obtained at each time point (l). Sedimentation rates of each experiment (m). (n–q) Growth of *bbs* cells cultured in KH medium for 7 days (n), biomass on Day 7. (o) Lipid (p) and paramylon contents (q). Error bars in all graphs show standard errors (*n* = 3). Significant differences were tested by one‐way ANOVA, and all statistical values were calculated by Dunnett's test; *P*‐values are shown.

Next, we assessed gravitational sedimentation of the non‐motile *bbs* mutants by time‐lapse imaging in 1‐min intervals (Figure 1j). We created 2D kymographs representing time‐dependent changes in the transparent–sediment interface level in *bbs* and WT cells, revealing the rapid sedimentation of the *bbs* mutants (Method S5, Figure 1k). We examined time‐series changes in the level of the transparent–sediment area in flasks segmented from binarized images. The WT cultures remained cloudy at 120 min after the flasks settled due to the presence of swimming cells, whereas *bbs* cells almost fully sedimented (Method S5, Figure 1l). Moreover, comparing the dry weights of *bbs* and WT sediments at 100 min after the flasks settled showed that the *bbs* mutants had 32–38% higher sedimentation rates than WT (Method S5, Figure 1m).

We also examined the growth, biomass, paramylon content and lipid content of WT and the *bbs* mutants (Method S6). Using the cell density of cells cultured in KH medium as estimate for growth, the growth rates of all four *bbs* strains were not significantly different from WT until 4 days after culture initiation, but the cell concentration of the bbs8 mutants was slightly higher than that of WT (p^bbs8‐B^ #4 = 0.042, p^bbs8‐D^ #13 = 0.011, Dunnett's test) at 7 days after culture initiation (Figure 1n). We did not detect marked differences between the *bbs* mutants and WT in terms of biomass harvested at 7 days after culture initiation or for the lipid content of cells under aerobic or hypoxic conditions (Figure 1o,p). Interestingly, the paramylon content of cells under aerobic conditions was significantly higher in the *bbs* mutants than in the WT (Figure 1q), which might be related to cellular motility and paramylon biosynthesis and/or accumulation. These results suggest that mutations in the *EgBBS* genes that facilitate sedimentation do not negatively affect the production of biomass or high‐value products.

During the mass cultivation of microalgae, harvesting accounts for 20–30% of total production costs; therefore, improving harvesting procedures will enhance the economic viability of microalgal biomass production (Brennan and Owende, 2010). We estimated the harvesting efficiency of the *bbs* mutants to be 32–38% higher than that of the WT (Figure 1m), while all strains produced similar amounts of biomass (Figure 1o, aerobic). Therefore, our results demonstrate that knocking out *EgBBS* genes in *E. gracilis* could improve harvesting efficiency without negatively affecting productivity.

## Conflict of interest

This study was partially supported by a matching fund‐based research programme between RIKEN and euglena Co., Ltd.

## Author contributions

MI, TN and ST contributed equally to this work. MI, TN, KY and ST conceived the study and designed the experiments. MI, TN, ST, KO, TS, TK and KH performed experiments and analysed the data. KM and KS supervised the project. KM and MI wrote the manuscript. All authors read and approved the final manuscript.

## Accession numbers

LC644194, LC644196–LC644198, LC644202 (Method S1).

## Supporting information


**Method S1** *Euglena gracilis* genome editing.
**Method S2** Genotyping of *bbs* mutants.
**Method S3** Microscopy observations.
**Method S4** Quantitative motion analysis.
**Method S5** Sedimentation analysis.
**Method S6** Growth, biomass, paramylon content, and lipid contents.
**Table S1** Oligos used in this study.
