# Supplementary material for: CRISPR/Cas9‐mediated generation of non‐motile mutants to improve the harvesting efficiency of mass‐cultivated Euglena gracilis
Source: Plant Biotechnol J. 2022 Sep 8;20(11):2042–4. doi: 10.1111/pbi.13904 (PMC9616515; doi:10.1111/pbi.13904)
Supplement: Supplementary file 1 — Method S1 Euglena gracilis genome editing. Method S2 Genotyping of bbs mutants. Method S3 Microscopy observations. Method S4 Quantitative motion analysis. Method S5 Sedimentation analysis. Method S6 Growth, biomass, paramylon content, and lipid contents. Table S1 Oligos used in this study. [file PBI-20-2042-s001.docx]

**Supporting Information**

**CRISPR/Cas9-mediated generation of non-motile mutants to improve the harvesting efficiency of mass-cultivated *Euglena gracilis***

Marumi Ishikawa^1,^ Toshihisa Nomura^1,2^, Shun Tamaki^1^, Kazunari Ozasa^3^, Tomoko Suzuki ^4,5^, Kiminori Toyooka^4^, Kikue Hirota^1^, Koji Yamada^1,6^, Kengo Suzuki^1,6^, and Keiichi Mochida^1,2,7,8,9^*

1. Microalgae Production Control Technology Laboratory, RIKEN Baton Zone Program, RIKEN Cluster for Science, Technology and Innovation Hub, Yokohama, Japan
2. Bioproductivity Informatics Research Team, RIKEN Center for Sustainable Resource Science, Yokohama, Japan
3. Advanced Laser Processing Research Team, RIKEN Center for Advanced Photonics, Wako, Japan
4. Mass Spectrometry and Microscopy Unit, Technology Platform Division, RIKEN Center for Sustainable Resource Science, Kanagawa, Japan
5. Center for Gene Research, Nagoya University, Aichi, Japan
6. euglena Co., Ltd., Tokyo, Japan
7. Kihara Institute for Biological Research, Yokohama City University, Yokohama, Japan
8. Graduate School of Nanobioscience, Yokohama City University, Yokohama, Japan
9. School of Information and Data Sciences, Nagasaki University, Nagasaki, Japan

*Corresponding Author: Tel: +81 (0)45 503 9111; Fax: +81 (0)45 503 9609; Email: keiichi.mochida@riken.jp

**Supporting experimental procedures**

**Methods S1 *Euglena gracilis* genome editing.**

We designed two independent pairs of single guide RNAs (sgRNAs) that target different regions of *EgBBS7* and *EgBBS8* genes (EgBBS7-A and -B; and EgBBS8- B and -D) and examined the DNA sequences of the genomic regions harboring each of their targeted sites. The genomic sequences of *EgBBS7* and *EgBBS8* have been deposited in DDBJ under the following accession numbers: LC644194, genomic sequence harboring EgBBS7-A Target site 1; LC644196, genomic sequence harboring EgBBS7-A Target site 2; LC644197, genomic sequence harboring EgBBS7-B Target site 1; LC644198, genomic sequence harboring EgBBS7-B Target site 2; LC644202, genomic sequence harboring EgBBS8-B Target site 1 and 2; and LC709282, genomic sequence harboring EgBBS8-D Target site 1 and 2.

We introduced Cas9 RNPs containing a pair of sgRNAs (5 µg each) synthesized with a CUGA 7 gRNA Synthesis Kit (NIPPON GENE) into *Euglena gracilis* Z strain provided by IAM (Tokyo, Japan) cultured in Koren–Hunter (KH) medium (pH 5.5) as described previously (Nomura *et al*. 2019). Following electroporation after a 24–72 h culture period in KH medium, a single cell was isolated with a micro pick-and-place system (Nepa Gene, JAPAN) as described previously (Nomura *et al.* 2020) and used to establish a mutant cell strain by culturing in KH medium.

**Methods S2 Genotyping of *bbs* mutants.**

Based on our previously published genome editing protocol (Nomura *et al*. 2020), genomic DNA template was extracted from the putative *bbs* mutant strains using a Kaneka Easy DNA Extraction Kit v.2 (Kaneka). DNA fragments were amplified with Tks Gflex DNA Polymerase using the specific primer set for each target site. The PCR products were cloned using a CloneJET PCR cloning kit (Thermo Fisher Scientific), and their sequences were determined by Sanger sequencing.

**Methods S3** **Microscopy observations.**

Microscopy observations of each *E. gracilis* strain were performed following culture in KH medium for 4 days. For bright-field microscopy, images of living *E. gracilis* cells were captured on a CKX53 inverted microscope (Olympus, JAPAN) connected to a DP27 camera (Olympus) with a 100x objective lens. For scanning electron microscopy, *E. gracilis* cells were fixed for 15 min by mixing in an equal volume of 2× glutaraldehyde solution (5% [w/v] GA, 100 mM cacodylate buffer pH 7.4). After removal of the fixing solution, 10% (v/v) ionic liquid (Hitachi High-Tech) was added, and the cells were immersed in the solution for at least 1 h. The treated cells were washed in deionized water and observed under a TM4000 scanning electron microscope (Hitachi high-tech) at an acceleration voltage of 5 kV, mode 2, and reflected electron setting.

**Methods S4 Quantitative motion analysis.**

*E. gracilis cells* (200−400 cells) grown in KH medium for 7 days were confined in a square microchamber (2.5 mm each side; 100 μm in depth). Motion analysis was performed as described by Muramatsu *et al*. (2020). The spatial sum of swimming trace pixels in the trace image was defined as trace momentum (Osaza *et al.* 2017).

**Methods S5 Sedimentation analysis.**

Sedimentation tests were performed using *E. gracilis* cells cultured in Cramer–Myers (CM) medium without sodium citrate and diammonium hydrogen phosphate (pH 3.5) for 7 days. A well-mixed 50-mL *E. gracilis* cell culture was placed in a culture flask and left to stand, and timelapse images were taken with a digital camera (PENTAX, K-S2) at one-minute intervals. The images were analyzed using ImageJ software (Abràmoff *et al*. 2004). Kymographs were created using one-pixel vertical lines extracted from the center of the flask area in the timelapse images. Transparent areas were also measured using images taken at ten-minute intervals converted to 8-bit grayscale and binarized based on the Otsu’s method (Otsu 1979), and the percentage of pixels derived from the transparent areas (supernatant) was determined in the culture medium areas (supernatant + sediment) cropped from the flask area.

Sedimentation rate was determined as follows:

$$Sedimentation rate \left( \% \right)=\frac{dry weight of sediment retrieved from the bottom 5ml after 100\min(g)}{dry weight of cells in 50ml placed in each flask at 0\min(g)}\times100$$

**Methods S6 Growth, biomass, paramylon content, and lipid contents.**

*E. gracilis* cells were seeded by adjusting the initial cell density to 8× 10^4^ cells/mL in KH medium (pH 3.5) and cultured for 7 days on a rotary shaker (120 rpm) at 29°C under 50 µmol m^−2^s^−1^ of light (12-h:12-h light-dark cycle). Cell density was measured with a particle analyzer (CDA-1000, Sysmex, JAPAN) each day. On the 7^th^ day, half of the culture was harvested by centrifugation (2,600 g, 5 min) for quantification of biomass. The other half was subjected to hypoxic incubation for 3 days, harvested, and analyzed as described for samples under aerobic conditions. The harvested cells were dried in a freeze dryer (FDV-1200, EYELA, JAPAN) to measure their biomass. Paramylon and lipid content were evaluated from the aerobic and hypoxic conditioned dry samples as previously described (Muramatsu *et al.*, 2020).

**References**

Abràmoff MD, Magalhães PJ, Ram SJ (2004) Image processing with ImageJ. *Biophotonics international* **11**: 36-42.

Muramatsu S, Atsuji K, Yamada K, Ozasa K, Suzuki H, Takeuchi T, Hashimoto-Marukawa Y *et al.* (2020) Isolation and characterization of a motility-defective mutant of *Euglena gracilis*. *PeerJ* **8**: e10002.

Nomura T, Inoue K, Uehara-Yamaguchi Y, Yamada K, Iwata O, Suzuki K, Mochida K (2019) Highly efficient transgene-free targeted mutagenesis and single-stranded oligodeoxynucleotide-mediated precise knock-in in the industrial microalga *Euglena gracilis* using Cas9 ribonucleoproteins. *Plant Biotechnology Journal* **17**: 2032-2034.

Nomura T, Yoshikawa M, Suzuki K, Mochida K (2020) Highly Efficient CRISPR-Associated Protein 9 Ribonucleoprotein-Based Genome Editing in Euglena gracilis. *STAR Protoc* **1**:100023.

Otsu N (1979) A threshold selection method from gray-level histograms. *IEEE transactions on systems, man, and cybernetics* **9**: 62-66.

Ozasa K, Won J, Song S, Tamaki S, Ishikawa T, Maeda M (2017) Temporal change of photophobic step-up responses of *Euglena gracilis* investigated through motion analysis. *PLoS One* **12**: e0172813.

**Table S1 Oligos used in this study**

| Name | Sequence (5’->3’) | Description |
| --- | --- | --- |
| CUGA7 crRNAtracrRNA | AAAAGCACCGACTCGGTGCCACTTTTTCAAGTTGATAACGGACTAGCCTTATTTTAACTTGCTATTTCTAGCTCTAAAAC | Oligo DNAs used to synthesize gRNA with the CUGA7 gRNA Synthesis kit |
| CUGA7 crRNAtracrRNA partial | AAAAGCACCGACTCGGTGCC |  |
| BBS7A1 | CTAATACGACTCACTATAGCGTGCCGGAGAAGGCGAAGGGTTTTAGAGCTAGAAATAGCA | Specific oligo DNA used to synthesize BBS7A target1 sgRNA |
| BBS7A2 | CTAATACGACTCACTATAGCAGCACGCTGGCCATCCTGAGTTTTAGAGCTAGAAATAGCA | Specific oligo DNA used to synthesize BBS7A target2 sgRNA |
| BBS7B1 | CTAATACGACTCACTATAGGGGCTTGGCCGGCTGCCCGGGTTTTAGAGCTAGAAATAGCA | Specific oligo DNA used to synthesize BBS7B target1 sgRNA |
| BBS7B2 | CTAATACGACTCACTATAGGCATCAACAGCATGGTGGTGGTTTTAGAGCTAGAAATAGCA | Specific oligo DNA used to synthesize BBS7B target2 sgRNA |
| BBS8B1 | CTAATACGACTCACTATAGGGCCACCTTGAAAGCCTGATGTTTTAGAGCTAGAAATAGCA | Specific oligo DNA used to synthesize BBS8B target1 sgRNA |
| BBS8B2 | CTAATACGACTCACTATAGGCGGACATCTGGTACAACATGTTTTAGAGCTAGAAATAGCA | Specific oligo DNA used to synthesize BBS8B target2 sgRNA |
| BBS8D1 | CTAATACGACTCACTATAGGGAGCGCCAGAGTGTCGGGGGTTTTAGAGCTAGAAATAGCA | Specific oligo DNA used to synthesize BBS8D target1 sgRNA |
| BBS8D2 | CTAATACGACTCACTATAGTCCGGAGCTCGAGGATGCCCGTTTTAGAGCTAGAAATAGCA | Specific oligo DNA used to synthesize BBS8D target2 sgRNA |
| BBA7A_F | ATCCAGGGCTCCTTCTCTCT | Primers for genomic PCR of *EgBBS7A* target region |
| BBS7A_R | GGCTCTTGACCTGCATCTTG |  |
| BBS7B_F | GAAGCAGAAGGTCGTCTTCG | Primers for genomic PCR of *EgBBS7B* target region |
| BBS7B_R | TCAGGCGACATGAAGAAATG |  |
| BBS8B_F | GGTTGGAATCCACAGAGATG | Primers for genomic PCR of *EgBBS8B* target region |
| BBS8B_R | GGCCAAGGACGACAACATG |  |
| BBS8D_F | AAGCGAGGGGTGAAAACAAC | Primers for genomic PCR of *EgBBS8D* target region |
| BBS8D_R | AAGGCGCTGGAGGTGTAC |  |
| pJET_F | CGACTCACTATAGGGAGAGCGGC | PCR primers for genotyping |
| pJET_R | AAGAACATCGATTTTCCATGGCAG |  |

Red letters indicate target sequences in each *EgBBS* target region.
